# Gender‐affirming hormone therapy: Effect on semen quality and use of fertility preservation

**DOI:** 10.1111/andr.70112

**Published:** 2025-08-21

**Authors:** Anna Chiara Conflitti, Alessandra Buonacquisto, Gaia Cicolani, Silvia Di Chiano, Marta Ruberto, Francesco Pallotti, Francesco Lombardo, Donatella Paoli

**Affiliations:** ^1^ Laboratory of Seminology ‐ Sperm Bank “Loredana Gandini”, Department of Experimental Medicine “Sapienza” University of Rome Roma Italy; ^2^ Department of Medicine and Surgery “Kore” University of Enna Enna Italy

**Keywords:** adolescence, cryopreservation, fertility preservation, spermatozoa, transgender, youth

## Abstract

Fertility preservation (FP) is a fundamental part of gender care. Gender‐affirming hormone therapy (GAHT) involves the use of drugs that could be potential risk factors for fertility. Therefore, FP is recommended before beginning the gender‐affirmation process. This narrative review aims to assess several aspects, mainly concerning the assigned male at birth (AMAB) population. The purpose of this review is to provide an overview of the effects of GAHT on testicular morphology and seminal quality, the use of semen cryopreservation, the desire for parenthood among transgender people, the factors influencing the decision to use fertility preservation (FP) and suggestions for improving clinical practice. A literature search within PubMed was conducted and the results deal with issues such as changes in testicular morphology and semen quality, FP utilization, barriers to treatment, current clinical practice models, and suggestions for improving transgender adolescents access to FP. The results showed that GAHT can have a negative effect on fertility in transgender AMAB subjects. It is therefore recommended to carry out FP before starting such treatment. However, there is low percentage of utilization to FP among transgender adolescents. In deciding whether to pursue FP, they consider many factors: future parenting desires, individual experiences of gender dysphoria, family values around biological parenting, financial considerations, and the availability of fertility information. A discrepancy emerged between the rate of desire to become a parent and the actual rate of FP utilization. The reasons are various, such as cost, and concern in delaying the initiation of treatment. Suggestions for improving clinical practice are also provided from this literature search.

## 1 INTRODUCTION

Gender incongruence is a marked and persistent incongruence between the gender experienced and the sex assigned at birth. The discrepancy between physical appearance and gender identity can lead to the need for medical and surgical intervention.^1^ Gender‐affirming hormone therapy (GAHT) involves the use of hormones to promote the development of desired sexual characteristics in people undergoing a gender‐affirmation process.^2^ In this narrative review, we focus on transgender individuals assigned male at birth (AMAB) for whom GAHT aims to reduce male secondary sex characteristics and induce female ones. Therapy typically involves the combined use of anti‐androgen drugs to block the effects of testosterone and estrogen to promote feminization.^3^ Of the available estrogens, 17‐β‐estradiol (both oral and transdermal) is the most commonly used. Among the anti‐androgen, cyproterone acetate (CPA) and spironolactone are the most commonly employed.^4^ During therapy, it is recommended to maintain serum estradiol and testosterone levels in the range typical of premenopausal women (100–200 pg/mL and <50 ng/dL, respectively).^2^ Modifications that occur include: reduction of facial and body hair, decreased libido and erections, increased breast size, and increased percentage of fat mass compared with muscle mass. However, these therapies can have side effects, including negative effects on fertility.^3^, ^4^ Professional organizations, such as the American Society for Reproductive Medicine (ASRM), the World Professional Association for Transgender Health, and the Endocrine Society, recommend counseling regarding the impact of medical treatment on fertility before starting GAHT or undergoing surgery. The potential options for fertility preservation (FP) include the cryopreservation of eggs, spermatozoa, or embryos.^2^, ^3^, ^5^ Cryopreservation of oocytes and spermatozoa is a feasible and effective method of preserving fertility for future biological parenthood and should be recommended to transgender people, especially adolescents.^6^ However, despite recommendations for access to FP, less than 5% of transgender people take this option.^7^, ^8^ The reasons for the low rates of FP use are not entirely clear. There is a lack of data regarding their desire to have biological children and their knowledge of family planning. In addition, transgender people may not be aware that FP options exist. Considering that the aim of this review was to explore the current state of knowledge regarding the impact of GAHT on fertility in transgender AMAB subjects, their desire to have or not biological children, their knowledge of the FP process, factors influencing the decision to use FP and current clinical practice models.

## 2 EFFECTS OF GAHT ON TESTICULAR MORPHOLOGY

Histological assessment of testicular morphology in transgender AMAB individuals undergoing gender‐affirmation surgery reveals heterogeneous patterns. Several studies have reported an approximately 25% reduction in testicular volume due to germ cell depletion and testicular atrophy within the first year of treatment.^9^, ^10^, ^11^ This feature is often accompanied by hyalinization of the seminiferous tubules^9^, ^11^ and a reduction^10^, ^11^, ^12^ or absence of Leydig cells.^9^, ^13^ Different types of drugs administered may have different effects, but the data are inconsistent. Due to a lack of information about the therapies used in some studies, we only reported the treatment when it was explicitly stated.

### 2.1 Ethinylestradiol

Treatment with ethinylestradiol has induced a testicular tissue phenotype characterized by the presence of only Sertoli cells and very few spermatogonia.^14^ In a different case, the same drug administered at varying concentrations caused absence of spermatogonia, absence of Leydig cells, hyalinization of seminiferous tubules, fibrosis, and testicular atrophy.^9^ Similar results were observed following therapy with ethinylestradiol combined with CPA: a transgender AMAB subject on this therapy for 18 months showed a decrease in the number and volume of Leydig cells, as well as arrest of spermatogenesis.^12^

### 2.2 Estrogens

Studies that have evaluated the effect of estrogens on testicular morphology have reported very different results. Some subjects exhibited normal spermatogenesis, while others showed altered spermatogenetic activity and depletion of Leydig cells.^15^ Schulze et al. examined testicular tissue from 11 patients treated with only estrogen (of varying type and dose) for 1–12 months. All histological specimens showed seminiferous tubules with only Sertoli cells and pale type A spermatogonia. Leydig cells were absent, but immature fibroblast‐like cells were present. Because estrogen can act directly on Leydig cells or indirectly through gonadotropin suppression, the authors hypothesized that the therapy caused dedifferentiation of Leydig cells into immature fibroblast‐like precursors^13^ In another study of five transgender AMAB subjects who underwent GAHT for 1–5 years, estrogen‐only therapy also resulted in the absence of Leydig cells. Only one patient showed normal testicular weight, although with impaired spermatogenesis. In all other cases, there was a reduction in testicular volume, testicular atrophy, and hyalinization of seminiferous tubules. Concerning spermatogenetic activity, the results were variable: none of the patients had spermatozoa, some presented spermatogonia, and others were devoid of germ cells.^11^ The effects of estrogen‐only therapy were also heterogeneous in two other papers, where some patients showed normal spermatogenesis and normal Leydig cell number, while others exhibited impaired spermatogenesis and depletion of Leydig cells.^16^, ^17^ Schneider et al. examined the testicular tissue of 108 transgender AMAB subjects treated with CPA in combination with estrogens, estrogens alone or spironolactone with estrogens. Histological evaluation was highly heterogeneous, independently of the type of therapy administered. Twenty‐four percent of the patients showed qualitatively normal spermatogenesis; the remaining had different patterns of altered spermatogenesis including meiotic arrest, spermatogonial arrest, and Sertoli cell‐only phenotype.^18^ In a more recent study, after 2 years of CPA and estrogen therapy, 75 out of 97 (77.3%) transgender AMAB subjects experienced spermatogenesis arrest. Of these, 12 (12.4%) had a Sertoli cell‐only phenotype and 63 (64.9%) had spermatogonial arrest. The remaining 22 subjects showed partial spermatogenesis: 8/97 (8.2%) reached secondary spermatocyte stage, while 14/97 (14.4%) arrested at the round spermatid stage.^19^ The difference in results in the two studies^18^, ^19^ may be explained by the different type of therapy and dosage. In the study by Vereecke et al., all participants were treated with CPA^19^; in contrast, in the study by Schneider et al., subjects were on different types of therapies with different dosages.^18^ Data in the literature to date suggest a heterogeneous clinical framework, with cases of spermatogenesis arrest, blockage at different maturational stages, or, in some cases, normal spermatogenesis.

The reason for the variability in response is still not completely clear. Some authors hypothesize that spermatogenetic activity depends on serum hormone levels. Specifically, in the study by Vereecke et al., serum testosterone levels appeared to influence the rate of sperm maturation: serum testosterone levels maintained in the female range resulted in suppression of spermatogenesis. In contrast, higher testosterone levels were associated with different germ cell maturative stages, although they did not go beyond the round spermatid stage.^19^ In addition to low serum levels of testosterone, high serum levels of estradiol are also associated with lower numbers of spermatogonia.^10^ Although none of the studies identified in our review directly investigated the specific effect of GAHT on local testosterone levels in the testes, it is important to consider that GAHT may affect testicular morphology and function by altering intratesticular testosterone concentrations. This suggests that further research is necessary to understand how hormone administration alters the testicular microenvironment, with potentially significant implications for fertility and how to provide gender‐affirming care for transgender AMAB people.

## 3 EFFECTS OF GAHT ON SEMINAL PARAMETERS

The main method of FP for transgender AMAB subjects is semen cryopreservation. Currently, there are few studies regarding who has benefited from this treatment, and data on semen quality before (Table 1), during (Table 2), and after therapy (Table 3) are limited.

**TABLE 1:** Publications that describe seminal parameters before therapy.

Author and publication year	Summary
Li et al.^22^	Retrospective study of 78 transgender assigned male at birth (AMAB) subjects compared with healthy cisgender (141). Transgender AMAB subjects showed poorer semen parameters both at the time of cryopreservation and post‐thawing.
Barnard et al.^25^	Retrospective study conducted on 10 transgender AMAB people. All patients presented semen parameters superimposable to reference values, with the exception of morphology.
Marsh et al.^23^	Case–control study composed of 22 transgender AMAB people. Sperm concentration, total spermatozoa per ejaculate, total motile spermatozoa, volume, and normal sperm morphology were significantly lower in transgender AMAB subjects compared with fertile cisgender men (17).
de Nie et al.^24^	Retrospective cohort study. The cohort consisted of 260 transgender AMAB subjects. Before gender‐affirming hormone therapy (GAHT), semen quality was significantly reduced compared with WHO (2010) data from the general population.
Rodriguez‐Wallberg et al.^20^	Prospective study of 177 transgender AMAB people. Subjects analyzed before GAHT showed lower seminal parameters than WHO (2010) reference values.
Sermondade et al.^26^	Retrospective study conducted on 83 transgender AMAB subjects. The semen parameters were not statistically different between transgender AMAB subjects had not started GAHT (*n* = 65) and sperm donors (38).
Amir et al.^27^	Retrospective study including 26 transgender AMAB adolescents. Semen quality and the outcomes of cryopreservation in adolescent transgender women are analyze. The semen quality is significantly lower compared with the WHO (2010) reference values.
de Nie et al.^21^	Prospective study conducted on a cohort of 113 transgender AMAB subjects. Seminal quality was below WHO (2010) reference values. Frequency of ejaculation, wearing tight undergarments and tucking have a negative impact on semen quality in transgender people

**TABLE 2:** Publications that describe seminal parameters during therapy.

Author and publication year	Summary
Jones et al.^32^	Retrospective study conducted on 9 transgender assigned male at birth (AMAB) subjects. Semen parameters were similar between those who were on gender‐affirming hormone therapy (GAHT; *n* = 6) and those who had never had therapy (*n* = 3).
Adeleye et al.^29^	Retrospective study conducted on 28 transgender AMAB people who underwent cryopreservation. The results show that subjects who were on therapy (*n* = 7) had poorer semen parameters than those who had never had GAHT (*n* = 18).
Sermondade et al.^26^	Retrospective study conducted on 83 transgender AMAB subjects. All semen parameters were significantly altered in transgender AMAB subjects on GAHT (*n* = 12) in comparison with those who had never had hormonal treatment (*n* = 65). Cyproterone acetate led to azoospermia in 100% of the cases (*n* = 6) in this series.

**TABLE 3:** Publications that describe seminal parameters after therapy.

Author and publication year	Summary
Leavy et al.^33^	Histological study conducted on nine transgender assigned male at birth (AMAB) subjects. Analyses of the testes obtained after orchiectomy showed promiscuous spermatogenesis with the presence of mainly spermatogonia.
Adeleye et al.^29^	Retrospective study conducted on 28 transgender AMAB people who underwent cryopreservation. The results show that after discontinuation of treatment (*n* = 3), semen parameters were similar to transgender AMAB subjects who had never used gender‐affirming hormone therapy (GAHT; *n* = 18).
Barnard et al.^25^	Retrospective study conducted on 10 transgender AMAB subjects. Two individuals who had already started GAHT showed transient azoospermia for 4–5 months after discontinuation of therapy.
Rodriguez‐Wallberg et al.^20^	Prospective study of 177 transgender AMAB people. Subjects with a previous history of GAHT (*n* = 16), lasting between 6 and 66 months and with discontinuation from 1 to 5 months, showed lower seminal parameters than subjects without previous GAHT (*n* = 161).
Sermondade et al.^26^	Retrospective study conducted on 83 transgender AMAB subjects. No differences were observed in transgender AMAB people who stopped GAHT (*n* = 5), in comparison with those who had never had GAHT (*n* = 65).

### 3.1 Semen parameters of transgender AMAB subjects before the initiation of GAHT

Some studies have suggested that semen parameters (total sperm count, sperm concentration, and total motility) of transgender AMAB people without therapy are significantly altered compared with WHO reference population^20^, ^21^ or cisgender men.^23^, ^24^, ^25^ In contrast, two cohort studies showed that the semen parameters of transgender AMAB people were comparable with those of fertile men, with the exception of morphology, which had a higher percentage of atypical forms compared with WHO (2010) reference values^25^ or a group of healthy donors.^23^ In addition, there is a high prevalence of azoospermia (1.8%,^21^ 3.1%,^26^ 4%^27^), oligozoospermia (15.4%,^26^ 16.8%,^21^ 28%^27^), oligoasthenoteratozoospermia (13.1%^21^), and asthenozoospermia (7.9%^21^) among transgender AMAB people without therapy, compared with the general population.^21^, ^26^, ^27^ The reasons for these alterations in the semen of this population, who are not undergoing treatment, are still unknown, but the causes could be attributable to lifestyle, such as the habit of wearing tight clothing or practicing tucking, a technique for hiding the testicles.^28^ These practices increase scrotal temperature and could negatively affect on testicular function and semen quality.^29^ Similarly, elevated scrotal temperature is known to impair testicular function and semen quality in cisgender individuals.^30^ Practicing tucking more than eight times a month or always wearing tight clothing significantly reduces total motile sperm count.^21^ Absence or decrease (less than six times per month) in ejaculation frequency can also cause a reduction in sperm production or progressive motility.^21^ Another possibility to consider is the self‐administration of estrogen, which often occurs without medical supervision and sometimes without being disclosed. In fact, as reported by Coleman et al., many patients present for care already undergoing hormone treatment, which may have been started independently, such as by purchasing medications online. This scenario implies the lack of adequate monitoring by a healthcare professional, carrying the risk not only of alterations in semen parameters, but also of potential side effects and harm to health.^31^

### 3.2 Semen parameters of transgender AMAB subjects during GAHT

It is possible that transgender people may not choose to preserve fertility before starting treatment, but they may decide to start during GAHT. The consequences of this therapy on seminal quality have been confirmed by several publications. Sermondade et al. reported that 12 transgender AMAB adults undergoing treatment with estrogen combined with progesterone or estrogen combined with spironolactone had significantly altered semen parameters when compared with a group of transgender AMAB subjects before starting therapy. The frequency of oligozoospermia (16.7%) and azoospermia (50%) was also higher during GAHT. In particular all subjects treated with CPA were azoospermic.^26^ Similarly, alterations in sperm concentration, motility, and percentage of atypical forms have been observed in transgender AMAB subjects on estrogen therapy (oral estradiol, transdermal estradiol, or conjugated estrogen) in combination with different anti‐androgens (spironolactone, finasteride, or micronized progesterone) for a median lengths of 30 months, compared with subjects who had not received therapy.^29^ In contrast, Jones et al. found no differences between the seminal characteristics of subjects on therapy and those who had never undergone therapy. In particular, the seminal parameter values of transgender AMAB subjects who had undergone feminization therapy for a relatively short time (median lengths = 0.33 months) were similar to those of the general population.^32^ Probably, the impact of GAHT on spermatogenesis depends on the nature of the drug administered, the duration of the therapy, and the dose administered.

### 3.3 Semen parameters of transgender AMAB subjects after discontinuation of GAHT

Many questions remain about the recovery of normal spermatogenesis after GAHT and the time window required. Some authors report recovery of spermatogenesis and improvement of semen quality after discontinuing hormonal treatment based on estrogen combined with anti‐androgens for 3–6 months.^26^, ^29^ The therapies were not the same for all subjects, the drugs used were oral estradiol, transdermal estradiol, or conjugated estrogen in combination with spironolactone, finasteride, or micronized progesterone. In particular, the semen parameters of transgender AMAB individuals who had discontinued therapy for 3–6 months were comparable to those who had never undergone GAHT.^26^, ^29^ Another study confirms that the prolonged use of estradiol‐based therapy (oral or transdermal) plus anti‐androgens (CPA or progestins) for a period of 1–6 years results in a complete arrest of spermatogenesis, with only the presence of spermatogonia in the seminiferous tubules.^33^ Rodriguez‐Wallberg et al. observed a significant decrease in sperm concentration and sperm motility in transgender AMAB subjects who had discontinued GAHT for 1–5 months, compared with a reference population of men whose fertility is unknown.^20^ Specifically, this study included 16 transgender AMAB subjects, six taking estrogen alone and 10 taking estrogen plus anti‐androgen.^20^ In the study conducted by Barnard et al., recovery of spermatogenesis was observed in a transgender AMAB subject 5 months after discontinuation of leuprolide acetate, which had been administered for 6 months. In contrast, a transgender AMAB individual on spironolactone and estradiol therapy was azoospermic up to 4 months after discontinuation.^25^ In another study, only CPA therapy caused azoospermia in all subjects, and none of them could benefit from FP anymore.^26^ These data suggest that recovery of spermatogenesis after treatment cannot be predicted and demonstrate the variability of the recovery. It is hypothesized that a discontinuation period of at least 3 months (a complete spermatogenesis cycle) is needed, but this could depend on the duration, dose, and types of hormones, as well as individual factors. In addition, even with the resumption of spermatogenesis, there are concerns about the potential effects of GAHT on the safety of male gametes and on embryo development and child health. These data show that subjects who cryopreserved semen before the start of GAHT have better semen parameters than transgender AMAB subjects in treatment at the time of sample collection. Particular attention should be paid to transgender AMAB adolescents before puberty, as they are unable to produce a semen sample.^34^ This may be a limitation for the use of FP in this population, who may be torn between the initiation of feminization therapy and its delay until puberty. However, research in this area could lead to the development of new techniques that would make FP possible before puberty.^34^ This scientific evidence supports recommendations to provide information on FP options prior to starting GAHT.

## 4 USE OF FERTILITY PRESERVATION AND DESIRE FOR PARENTHOOD

Some recent studies have reported that, for young transgender people, fertility is not a priority.^32^, ^33^ Chiniara et al. performed a questionnaire‐based cross‐sectional study that assessed knowledge of the potential effects of GAHT on fertility, present and future life priorities, and preferences regarding future fertility/parenthood options among young transgender individuals and their parents. All participants knew that GAHT could alter fertility, but none of them chose to proceed with FP. The most important current life priority for youth was being healthy, and the least important priority was having children.^35^ In fact, in studies conducted to date, less than 5% of transgender adolescents have chosen FP.^7^, ^8^ However, there seems to be a discrepancy between the number of transgender adolescents who want to have children and those who choose FP. The majority of young transgender subjects say they would like to become parents in the future, but most do not plan to have a biological child: they prefer adoption or foster care,^35^, ^36^, ^37^ and a smaller number are open to surrogacy or gamete donation.^35^, ^38^ The reasons for this difference are related to social, financial, biological, and ethical implications of the use of assisted reproductive technologies (ART), that complicate the decision‐making process toward future biological parenthood for transgender adolescents.^35^, ^39^, ^40^

### 4.1 Barriers for patients

There are several reasons why transgender people prefer an alternative family to a biological one. The most commonly cited barriers are cost and invasiveness.^8^, ^40^, ^41^, ^42^ Some studies report that the rate of cryopreservation in transgender AMAB people is higher than subjects assigned female at birth (AFAB).^7^, ^8^ This evidence is not surprising considering that the cost and invasiveness of procedures differ according to the sex assigned at birth. Semen cryopreservation is less expensive than oocyte cryopreservation. Furthermore, the process of gender‐affirming care for AFAB typically involves more complex procedures, such as ovarian stimulation and egg retrieval, compared with semen collection via masturbation.^42^ For transgender people we also find unique barriers that may affect FP utilization rates, such as gender dysphoria, discomfort with gamete retrieval procedures, and concerns about delayed GAHT. Transgender people who present to gender clinics often have a need to undertake hormonal intervention immediately and consequently are reluctant to delay the start of therapy.^8^, ^35^, ^40^ The desire for parenthood is also influenced by gender identity and gender role. A study has revealed that some young transgender people feared body dysmorphia associated with pregnancy; some worried that undergoing fertility treatments in line with their sex assigned at birth would disrupt their process and exacerbate gender dysphoria; some described themselves as too young to make a decision about fertility; and some would have liked to cryopreserve but feared that their choice might cause family and friends not to truly believe in their gender incongruence.^36^ The need to masturbate was another reason for refusing FP. Alternative sperm extraction options such as sperm extraction from testis or electroejaculation stimulation could be used to facilitate collection.^7^, ^8^ The desire for parenthood is also influenced by gender identity and gender role: transgender youth feel it is important to experience parenthood through the role associated with their gender identity and not their assigned sex at birth.^40^ It should also be taken into account that people's interest in FP may be influenced by their sexual orientation which could require the use of donor eggs and/or a gestational surrogate.^35^, ^41^

### 4.2 The influences of parents

Decisions to start GAHT are often made before the age of 18, so parental involvement and consent is required in FP discussions. The role of parental wishes can impact children's decision, but it must be recognized that these decisions can be difficult for parents to make with or for their children. Persky et al. administered a survey to parents and children to understand their thoughts and factors influencing FP. Although most youth and their parents reported that they had considered the impact that GAHT may have on fertility (69% youth and 83% parents), most of them did not think it was important to have biological children or grandchildren, respectively. Moreover, 69% of youth and 63% of parents said that they or their child would be able to decide at this age. This study found that parents tend to think similarly to their children's wishes and are understanding about their decisions.^43^ Similarly, Chiniara et al. reported that priority rankings are similar between parents and sons: most parents (65%) considered having children today to be one of the lowest priorities. However, having children seemed to gain importance in 10 years for both transgender youth and their parents. Despite, parents' and children's views on FP do not always converge, and this can have an impact on decision‐making.^35^

### 4.3 Decision‐making regret

A growing number of research suggests that transgender adults desire biological parenting.^44^, ^45^ The difference between the desires of the adolescent and adult population raises the question of whether these transgender youth may change their perspectives on FP later in life.^8^ The survey conducted by Vyas et al. showed that transgender people who had chosen not to pursue FP or who had been undecided in the past subsequently experienced mild to severe regret. This discordance between patients' interests at the first visit and their regret during follow‐up shows that ideas can change. This may be because the average age of patients at baseline assessment is lower than that of the follow‐up group; consequently they may have had more time to reconsider their position on FP or may have found that priorities shifted as they reached later stage of life.^45^ As a result, good counseling could support transgender youth in the decision‐making process and help avoid future regrets.

### 4.4 Lack of counseling

Decision‐making can be influenced by the supportive conversations transgender youth receive from their providers. In the study by Boguszewski et al., many participants emphasized the importance of collecting accurate and trans‐specific information in order to make a conscious decision about FP. Some transgender youth expressed frustration because they did not receive more help directly from their healthcare providers, while others were not satisfied with the information they obtained. In addition, participants in this study noted that the information they were given about FP options did not reflect all parenthood options.^40^ The possibility of planning a family with a partner who is biologically the same sex as the transgender person, thus having the same genitalia, is often not discussed. This consideration is important for FP because it may influence the choice between having biological children or adopting, which is why transgender people want more information and guidance on family planning. The first question is: who should provide this information? Quain et al. conducted a study that aimed to examine the preferences of transgender adolescents and their parents regarding the provision of FP information and timing of discussion. Most survey participants (88%) preferred gender clinic physicians to provide information about FP, while a smaller portion preferred fertility specialists (23%) or mental health professionals (28%). Therefore, gender clinicians, mental health professionals, and fertility specialists should be prepared to discuss FP and provide appropriate counseling. One unexpected finding was that two participants cited parents as an additional source of FP information. Although parents are not FP experts, this finding underscores the importance of involving parents in FP decision‐making.^46^ This aspect also emerged in another study, in which many young participants described the opportunity to reflect on their future fertility in a conversation with a family member or friend, as important for contextualize their decision and setting their priorities.^40^ The second question is: when should FP be discussed? The survey conducted by Quain et al. found there is no universal ideal timeline, but discussion times must be tailored to each patient. Physicians should evaluate the amount of information provided during the first clinical visit to determine whether to discuss FP at the initial meeting or wait for future visits. Alternatively, clinicians may briefly mention FP during the initial visit and allow families time to consider it before a more in‐depth discussion at a future appointment.^46^

## 5 BARRIERS FOR PROVIDERS

Although FP counseling is recommended by the World Professional Association of Transgender Health (WPATH), the American Society of Reproductive Medicine (ASRM), and the Endocrine Society, there are no currently guidelines available for specialists performing fertility counseling in transgender people. The absence of such guidelines is a barrier for providers who should appropriately advise patients and families and approach various complexities with them. Similar to oncofertility, studies have shown that gaps in provider knowledge about the impact of antineoplastic therapies on fertility have led to low utilization,^47^ and that more standardized counseling results in higher FP rates.^48^ It is therefore important to explore the current behaviors and knowledge of providers tasked with counseling transgender people and their families about FP, especially considering the risks in this population for future decision regret regarding potentially irreversible choices. Chen et al. examined fertility knowledge and practice behaviors among multidisciplinary providers caring for transgender subjects. More than 80% of respondents recognize that exogenous hormones can decrease fertility, and 95% accurately know WPATH and Endocrine Society recommendations for fertility counseling prior to hormonal interventions. Despite this, less than half of the participants admitted to recommending patients to fertility specialists.^49^ In contrast, in the study of Tishelman et al., many providers stated that they have gaps in knowledge about FP. For example, they are not well‐informed about the fertility risks of various hormonal treatments, which FP options are appropriate, or the success potential of preservation.^42^ It is important to provide the right information to the patient to avoid them turning to the Internet or friends with the risk of being inaccurately informed.^48^ Providers commented on the need for general guidelines in order to standardize counseling practices and counsel patients more effectively.^42^

## 6 HOW TO IMPROVE CLINICAL PRACTICE

Fertility counseling for transgender people should address the potential risks associated with GAHT and its effects on long‐term fertility. Studies suggest that counseling should provide evidence‐based information that includes knowledge of the known effects of hormones, risks and implications for reproductive health. This approach could help reduce the risk of regretful decisions in the future. In the study by Lai et al., clinicians believed that a multidisciplinary team was important for the efficacy of fertility counseling. Having a multidisciplinary team within the same clinic improves the quality of care through the collaboration of clinicians who specialize in different disciplines. In addition, it allows for the sharing of expert advice and information immediately among various clinicians.^50^ A recommended minimum team composition should include specialists in endocrinology, urology, pediatric surgery, fertility medicine, genetic counseling, and mental health.^49^ Physicians recognized that an important part of the decision‐making process was giving people time to make their decisions consciously. In this regard, the provision of written information could be helpful, allowing patients to review the information on their own time.^50^ Chen et al. also argued that verbal counseling alone may not be enough, so creating written information brochures that patients can take home and consult at their leisure would be helpful.^49^ Lai et al. also emphasized patient involvement. An important factor during conversations is language because it is often difficult to sustain a conversation on these issues without causing discomfort. These themes could trigger gender dysphoria or be seen as a potential impediment to a transgender person's desire to immediately begin GAHT. In addition, it would be appropriate to train staff in the clinics (e.g., nurses) to avoid misgendering.^50^ Another aspect that could be improved in clinical practice is related to care. Physicians have suggested that personalized care is important to meet the specific needs of patients.^50^ Chen et al. also highlighted the need to personalize the approach and discuss these complex issues with transgender youth in an age‐appropriate way.^49^ In addition, the development of a decision aid could improve patient–clinician communication, ensure that the entire multidisciplinary team delivers a consistent message, reduce decision‐making conflicts, and increase satisfaction with decisions.^50^, ^51^

## 7 CONCLUSION

### 7.1 Testicular morphology

GAHT may induce changes in testicular morphology such as reduction or absence of germ cells, absence of Leydig cells and hyalinization of the seminiferous tubules, although it does not always cause alterations in testicular function. In fact, studies conducted so far have identified variable clinical patterns: arrest of spermatogenesis at different maturation stages, or normal spermatogenesis.

### 7.2 Semen quality

Data in the literature indicate that transgender AMAB subjects before the initiation of GAHT have better seminal quality than subjects who have discontinued hormone treatment or are on therapy at the time of semen evaluation. However, although spermatogenetic activity may be altered, subjects undergoing GAHT are not always azoospermic. The use of contraception is therefore recommended in order to avoid undesired pregnancies. The length of the time window required for recovery of spermatogenetic activity after treatment discontinuation is unknown. A minimum of 3 months is considered necessary, but there are documented cases where azoospermia has persisted for up to 5 months. Therefore, recovery of spermatogenesis depends on the duration and dose of therapy, as well as individual factors.

### 7.3 Timing of semen cryopreservation

Cryopreservation of semen should always be recommended before GAHT because the recovery time of spermatogenesis and the safety of these gametes are not yet known.

### 7.4 Cryopreservation decision

Although recent studies have found that most transgender subjects are informed about the risk of infertility caused by GAHT, a low rate of use of semen cryopreservation in transgender AMAB subjects has been reported. The main barriers are cost, invasiveness, and the impossibility of delaying or stopping treatment. These barriers could be overcome by improving FP counseling. This could be achieved by setting up a multidisciplinary team; developing specific training courses for healthcare professionals so that they are aware of the possibilities of FP; providing clear, detailed, and preferably written information; and personalizing the approach according to the clinical situation of each patient.

## AUTHOR CONTRIBUTIONS

Donatella Paoli and Anna Chiara Conflitti designed this review paper. Anna Chiara Conflitti, Alessandra Buonacquisto, Gaia Cicolani, Silvia Di Chiano, and Marta Ruberto conducted a literature search and collected the data. Donatella Paoli performed the final screening, determined the relevance of the articles, and resolved any disagreements. Anna Chiara Conflitti wrote the manuscript and prepared the tables. Francesco Pallotti, Francesco Lombardo, and Donatella Paoli revised the manuscript. All the authors have read and approved the final version of the manuscript.

## CONFLICT OF INTEREST STATEMENT

The authors declare no conflicts of interest.

## Data Availability

The data that support the findings of this review are included in this published paper.
